# Editorial

**Published:** 1876-04

**Authors:** 


### Editorial.

We are sorry to see that the *Medical Press and Circular*, a worthy English cotemporary, has been the subject of gross imposition by its "American Correspondent." A few quotations will show the spirit of the whole communication.

The correspondent sends the following about Chicago: "The Medical Colleges of this city are now holding their commencements. In other words, they are now in the act of sending forth their annual quotas of legitimized murderers to butcher and poison the human race. * * It is said that one of the medical colleges has never plucked a student, every one who paid received his diploma. * * In the interests of the health of the people every medical college should be indicted—and its doors closed. * * The facility with which degrees can be obtained is extensively taken advantage of by clerks, artisans, etc. I am *credibly* (?) informed by independent, disinterested, trustworthy persons, that dry-goods clerks, post-office clerks, letter carriers, etc., buy a book or two and study at home of evenings. When these parties believe they know the book sufficient to answer by rote a simple common-place question or two of a professor in one of the colleges, they appear before the examiners and of course pass. * * *

"The medical profession of Chicago is become vile. Physicians are not respected. Why should they be? They are, with few exceptions, shamefully, fearfully, criminally ignorant. They are ever making the grossest blunders in practice. * * * We have been assured, and our own experience confirms the assertion, that there are men practicing medicine in this city (Chicago) for periods ranging between one year and twenty years who could not tell where the human heart is situated."

These are a few extracts from a communication in the

*Medical Press and Circular*, Dec. 29, 1875. They will give the reader a pretty good idea of its contents. We do not expect to notice the communication as it might very properly be noticed ; because we feel mortified and not angry, that it could by any possibility have found its way into a respectable medical journal. We are not ashamed of the revelations made in the letter, because there is no truth in them, and we have too much respect for the good sense of the editor of the *Medical Press and Circular* to entertain in the remotest manner the idea that he believes one of the quotations above made, to be applicable to the regular profession of Chicago. Believing this much, have we not reason to be mortified that an influential medical periodical should even unwittingly lend its pages to the vile use of falsifying and consequently injuring the profession by which it is supported ?

Invectives seldom embody truth—they are born of envy and hate, and while hot with anger they consume the subject from which they emanate, their exaggeration is too easily discovered to injure the object at which they are aimed. When they are found in a medical journal they are out of place, and especially when their object is the medical profession.

There are three medical colleges here : Rush Medical College, Chicago Medical College, and the Woman's Hospital Medical College. We can assure the editor of the *Medical Press and Circular* that the quotations above made are not applicable to either of these institutions, and that his correspondent has not only imposed upon him, but we have good reason to believe has been grossly imposed upon himself. If the correspondent has visited Chicago he has not associated with the better class of those practicing medicine here ; he has kept bad company, and lacks the discrimination necessary to keep him out of it.

The correspondent indulges in *personal* abuse in one instance, and in doing so, gets as far from the truth as in the above quotations. In speaking of the selection of professors, he says, "A good example is afforded by the Woman's Medical College of Chicago, where one Sarah Hackett Stevenson, immediately after graduation *at the end of twenty-one weeks of study*, is appointed to the chair of physiology."

Mrs. Stevenson did not graduate until the end of three full years of study, one of which was spent in the schools of Europe, and a good part of the time under the tuition of Huxley and Tyndall.

Many of the quotations made in this editorial, were by the correspondent derived from the Chicago *Times*. This does not lessen his responsibility for them, as he ought to have been sure of their truth before he endorsed them with such venomous recklessness. We must say, in conclusion, that the *Medical Press and Circular* has placed itself in a false position before the profession by admitting such a base fabrication to appear in its pages, and we believe it to be the duty of its editor to purge himself of complicity in this ungentlemanly attack upon the medical institutions and members of the profession in Chicag[illegible]

